# Methylation plotter: a web tool for dynamic visualization of DNA methylation data

**DOI:** 10.1186/1751-0473-9-11

**Published:** 2014-06-07

**Authors:** Izaskun Mallona, Anna Díez-Villanueva, Miguel A Peinado

**Affiliations:** 1 Institute of Predictive and Personalized Medicine of Cancer (IMPPC), Ctra. de Can Ruti, camí de les escoles, s/n, 08916 Badalona, Spain; 2 Health Research Institute Germans Trias i Pujol (IGTP), Ctra. de Can Ruti, camí de les escoles, s/n, 08916 Badalona, Spain

**Keywords:** Methylation plot, Methylation visualization, R/shiny, Lollipop plot

## Abstract

Methylation plotter is a Web tool that allows the visualization of methylation data in a user-friendly manner and with publication-ready quality. The user is asked to introduce a file containing the methylation status of a genomic region. This file can contain up to 100 samples and 100 CpGs. Optionally, the user can assign a group for each sample (i.e. whether a sample is a tumoral or normal tissue). After the data upload, the tool produces different graphical representations of the results following the most commonly used styles to display this type of data. They include an interactive plot that summarizes the status of every CpG site and for every sample in lollipop or grid styles. Methylation values ranging from 0 (unmethylated) to 1 (fully methylated) are represented using a gray color gradient. A practical feature of the tool allows the user to choose from different types of arrangement of the samples in the display: for instance, sorting by overall methylation level, by group, by unsupervised clustering or just following the order in which data were entered.

In addition to the detailed plot, Methylation plotter produces a methylation profile plot that summarizes the status of the scrutinized region, a boxplot that sums up the differences between groups (if any) and a dendrogram that classifies the data by unsupervised clustering. Coupled with this analysis, descriptive statistics and testing for differences at both CpG and group levels are provided.

The implementation is based in R/shiny, providing a highly dynamic user interface that generates quality graphics without the need of writing R code. Methylation plotter is freely available at http://gattaca.imppc.org:3838/methylation_plotter/.

## Background

Cytosine methylation in CpG dinucleotides is an important mechanism involved in the regulation of multiple biological processes including pathological conditions [1-3]. While there is a wide range of methodologies to evaluate DNA methylation, bisulfite-treated DNA sequencing is the gold standard to determine DNA methylation at the single CpG level [1,4,5]. The functional implications of DNA methylation states are often determined by the CpG profile but at the regional level rather than by a single CpG site. Therefore, the interpretation and application of this sort of data require further analysis that is highly benefited by the implementation of visualization tools.

While some software tools to analyze and visually represent DNA methylation data have been published (reviewed in [5]), its use by wet lab users is often limited. On the other hand, popular spreadsheet tools like Excel are unable to generate lollipop plots by default. Even more, the Excel-based solutions perform poorly for repetitive tasks: in an automated analysis context, programmatic approaches are less error prone and more reproducible [6].

Specialized tools have been developed to work with converted bisulfite sequence files and to explore methylation trends, but are highly dependent on the operating system: MethTools, [7]) is Unix-based, and CpG Analyzer [8] or CpG PatternFinder [9]) run under Windows. MethDB [10] offers a web tool and thus is platform-independent, but is designed as a methylation data provider rather than a graphical tool. BiQ Analyzer [11,12] and QUMA [13] provide web tools that plot lollipop-like graphics; however, they are rather devoted to clonal analysis, assessing the methylation status as a categorical variable (either methylated or unmethylated). Hence, a platform-independent tool to visualize continuous methylation data, as those produced by direct bisulfite sequencing or microarray platforms, is needed.

## Implementation

The interactive web application is written using *shiny*, an R framework that couples the R-based statistics computation and graphics generation to the rendering of a Web-based user interface [14]. This technology allows to take advantage of the R power in an easy-to-use frontend. As the application is hosted in a remote server, the user does not need to consume local resources and just requires a Web browser to use the tool. User data is removed from the server as soon as the browser session terminates.

## Results and discussion

Methylation plotter is an interactive application that allows rapid and easy generation of customized plots and statistical summaries of methylation data. The user is asked to upload a tab-separated file describing the status of up to 100 CpGs in up to 100 different samples as well the group each sample belongs to. The application generates an interactive plot that summarizes the status of every CpG site and for every sample in lollipop or grid styles. Methylation values ranging from 0 (unmethylated) to 1 (fully methylated) are represented using a gray color gradient.

**Figure 1:**
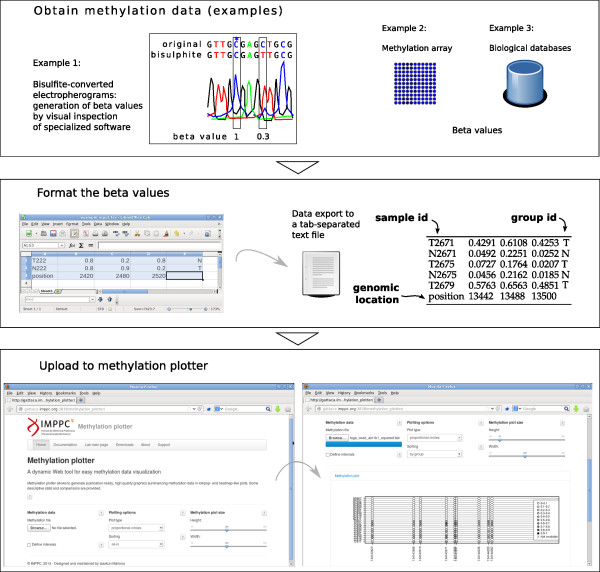
**Data input and usage flowchart.** Methylation plotter uses beta-values as input. These can be obtained directly from methylation array platforms such as the Illumina Infinium 450k, downloaded from databases like the TCGA or from bisulfite-treated DNA sequencing. For instance, direct bisulfite sequencing provides an estimation of the beta-value of each cytosine. In this case, the C to T peak height ratio can be assessed by naked eye and reflects the methylation status of that position. Once obtained the beta values, the user may use a spreadsheet editor (Microsoft Excel, LibreOffice Calc) to format the data and to export it to a tab-separated text file. Finally, the upload of this file to the webpage will produce the methylation plot and the rest of graphical and statistical outputs. The plotting options (data sorting, plot type, image width and height) are dynamically changed without the need of reuploading the data.

**Figure 2:**
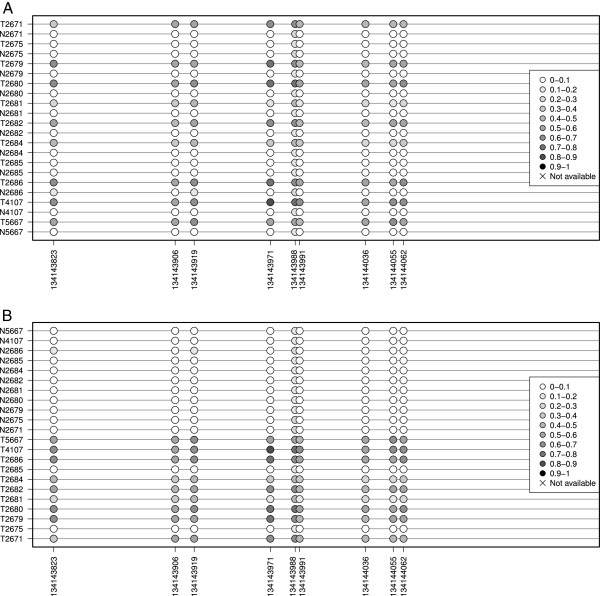
**Lollipop-like visualization with Methylation plotter.A**, the input data alternates normal and tumor tissue data. **B**, data visualization after explicitly sorting the samples according to the tissue type; the pattern of tumor hypermethylation is easily detectable.

**Figure 3:**
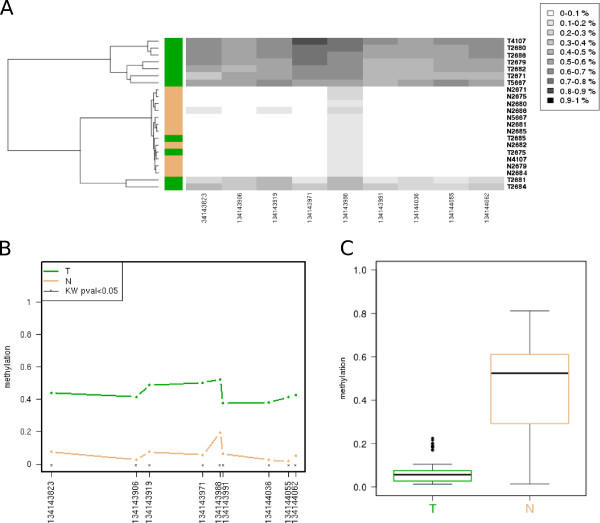
**Data visualization with Methylation plotter.A**, unsupervised hierarchical clustering of the data; sample label colours reflect the user-provided classification. **B**, methylation profiling plot reflecting with asterisks those positions for which significant differences between groups were detected. **C**, boxplots for each group showing the methylation data distribution.

The input data consist on beta values, a popular format, that offer an intuitive manner to represent the level of methylation. These beta values are typically generated by the software used to process bead arrays like the Illumina Infinium HumanMethylation450 [15]. Data portals such as the The Cancer Genome Atlas (TCGA) provide beta-values in a comprehensive series of cancer genomics datasets. However, wet lab users oftenly perform bisulfite-treated sequencing of their samples, and therefore require further preprocessing in order to assess the methylation status. For instance, an electrophoregram viewer or even a sequence alignment tool may be necessary. A flowchart of the data acquisition and processing steps is available as Figure 1. An excellent outline of the bisulfite data preprocessing may be found at [11].The methylation plot is interactive: without the need of reuploading the data, the user can customize the plot dimensions on the fly and therefore produce publication- ready figures. Accordingly, the user can select different types of arrangement of the samples in the display: for instance, sorting by overall methylation level, by group, by unsupervised clustering or just as provided. Finally, the lollipop plot allows to select whether to keep the distances between CpGs proportional (that is, disregarding the actual distance) or not. Figure 2 shows a typical lollipop-like output plot, as well the by-group sorting (Figure 2B). For bulky datasets, the user can select a more convenient heatmap-like plot that represents all the scrutinized CpGs in a grid-like manner.Beyond the lollipop or grid-like methylation plots, the tool provides three data representations. First, a heatmap with its associated dendrogram offers the result of the unsupervised clustering of the samples, colouring each dendrogram leaf according to the user-provided group (Figure 3A); this allows an easy checking of coherence between the already established groups and those generated by the unsupervised classification. Second, a profile plot summarizes the methylation panorama according to the sample group, labelling those CpGs that show statistical differences according to the nonparametric test Kruskal-Wallis (Figure 3B). And third, a boxplot depicts the methylation profile for each group highlighting its quartiles, thus simultaneously summarizing the methylation status for each group of samples (Figure 3C).

Altogether, Methylation plotter provides descriptive statistics and basic non-parametric variance analysis (Kruskal-Wallis tests). For each sample, a data table summarizing the mean, standard deviation, minimum and maximum, and number of not available positions (NAs) is produced. The same descriptive statistics are produced for each CpG and, if the input data is ascribed to two or more groups, each CpG is tested for equality using the Kruskal-Wallis test.

All the figures are available to download as either raster (PNG) or vector format files (PDF), whereas statistical reports are served as spreadsheets (tab-separated values).

## Conclusions

In summary, Methylation plotter is a user-friendly tool that produces ready-to-use plots and summary data required by most wet lab users analyzing DNA methylation. The application is freely accessible at http://gattaca.imppc.org:3838/methylation_plotter/.

## Availability and requirements

•Project name: Methylation plotter

•Project home page: http://sourceforge.net/projects/methylationplotter

•Operating system(s): Platform independent

•Programming language: R/shiny

•Other requirements: None

•License: GPL v2

•Any restrictions to use by non-academics: None

## Competing interests

The authors declare that they have no competing interests.

## Authors’ contributions

IM, ADV and MAP conceived the project. IM and ADV implemented the software using R code. IM designed and coded the Web tool. IM wrote the manuscript and all the authors read and approved it.
